# Three classes of ligands each bind to distinct sites on the orphan G protein-coupled receptor GPR84

**DOI:** 10.1038/s41598-017-18159-3

**Published:** 2017-12-20

**Authors:** Zobaer Al Mahmud, Laura Jenkins, Trond Ulven, Frédéric Labéguère, Romain Gosmini, Steve De Vos, Brian D. Hudson, Irina G. Tikhonova, Graeme Milligan

**Affiliations:** 1 Centre for Translational Pharmacology, Institute of Molecular, Cell and Systems Biology, College of Medical, Veterinary and Life Sciences, University of Glasgow Glasgow, G12 8QQ Scotland, United Kingdom; 2 Department of Physics, Chemistry and Pharmacy, University of Southern Denmark, Campusvej 55, DK-5230 Odense M, Denmark; 3 Galapagos SASU, 102 Avenue Gaston Roussel, 93230 Romainville, France; 4 Galapagos NV, Generaal De Wittelaan L11 A3, 2800 Mechelen, Belgium; 5 School of Pharmacy, Medical Biology Centre, Queen’s University Belfast, Belfast, BT9 7BL United Kingdom; 6 Present Address: Evotec, 195 Route d’Espagne, 31100 Toulouse, France

## Abstract

Medium chain fatty acids can activate the pro-inflammatory receptor GPR84 but so also can molecules related to 3,3′-diindolylmethane. 3,3′-Diindolylmethane and decanoic acid acted as strong positive allosteric modulators of the function of each other and analysis showed the affinity of 3,3′-diindolylmethane to be at least 100 fold higher. Methyl decanoate was not an agonist at GPR84. This implies a key role in binding for the carboxylic acid of the fatty acid. Via homology modelling we predicted and confirmed an integral role of arginine^172^, located in the 2nd extracellular loop, in the action of decanoic acid but not of 3,3′-diindolylmethane. Exemplars from a patented series of GPR84 antagonists were able to block agonist actions of both decanoic acid and 3,3′-diindolylmethane at GPR84. However, although a radiolabelled form of a related antagonist, [^3^H]G9543, was able to bind with high affinity to GPR84, this was not competed for by increasing concentrations of either decanoic acid or 3,3′**-**diindolylmethane and was not affected adversely by mutation of arginine^172^. These studies identify three separable ligand binding sites within GPR84 and suggest that if medium chain fatty acids are true endogenous regulators then co-binding with a positive allosteric modulator would greatly enhance their function in physiological settings.

## Introduction

**Figure 1:**
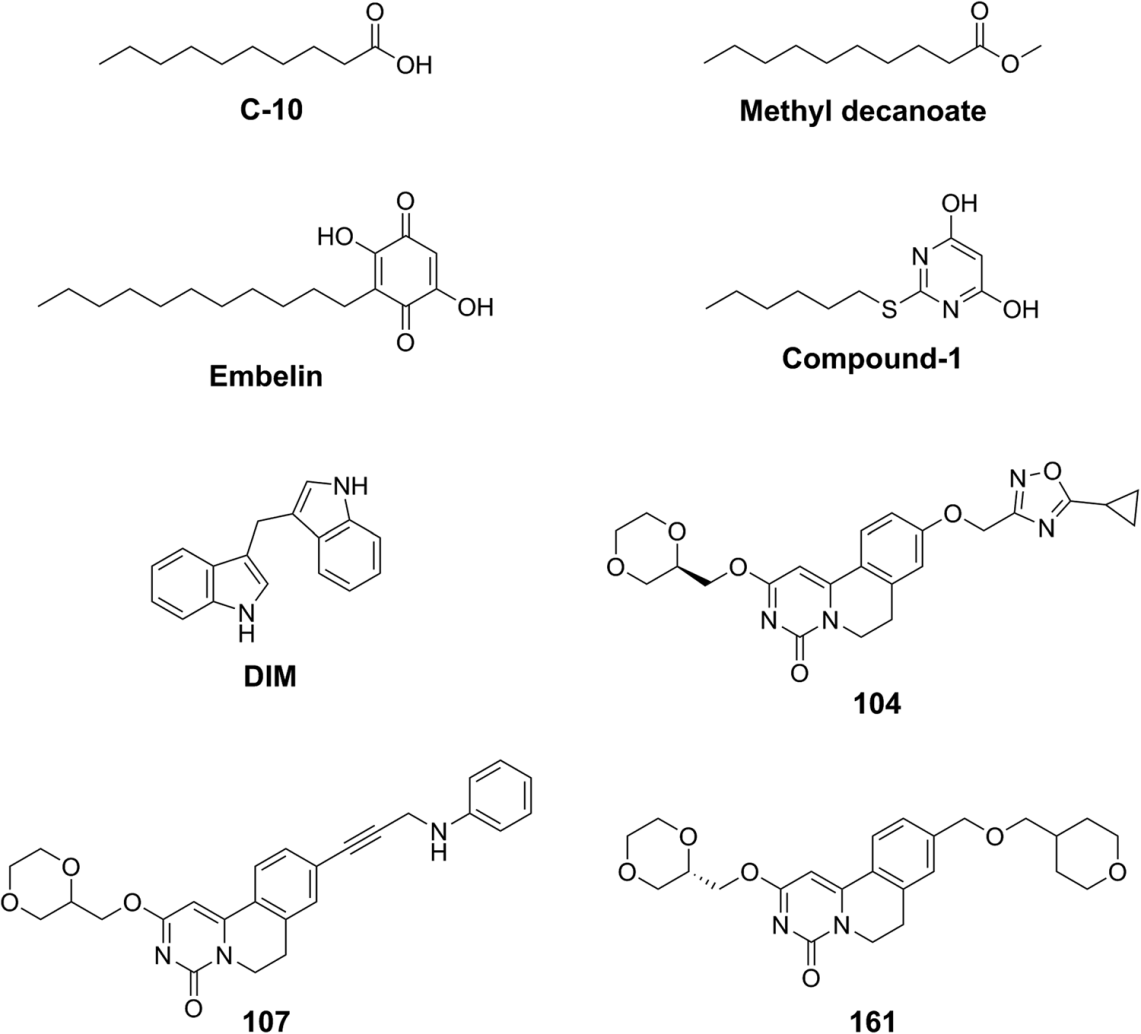
Structures of GPR84 ligands used in the studies. Chemical structures of the MCFA C10, the equivalent methyl ester, a pair of synthetic GPR84 orthosteric agonists (embelin, compound-1), the allosteric GPR84 agonist DIM and three exemplar GPR84 antagonists (compounds 104, 107 and 161) are shown.

GPR84 is designated officially as an ‘orphan’ G protein-coupled receptor (GPCR)^1^. This terminology indicates that the endogenous ligands that activate the receptor remain unidentified or that suggested ligands are not accepted with unanimity by the research community. Further work is needed, therefore, to validate suggested pairings. Despite this, the ability of medium chain fatty acids (MCFAs) to act as activators of GPR84 was first reported more than 10 years ago^2^. A number of subsequent studies have confirmed the ability of MCFAs, and also of hydroxylated MCFAs, to activate GPR84^3–6^, although this has not been observed in all studies^7^. The most potent of the MCFAs in activating GPR84 are generally decanoic (also called capric) acid (C10) (Fig. 1) and undecanoic acid (C11). Lack of full acceptance of MCFAs as the key endogenous activators of GPR84 may reflect that the mode of interaction of the MCFAs with GPR84 is poorly defined^3,8,9^ and that the reported potency of these ligands at GPR84, particularly in cells natively expressing this receptor, is very modest^2^.

All GPCRs that are currently accepted by the International Union of Basic and Clinical Pharmacology to be receptors for fatty acids contain either a single, (free fatty acid receptor 4, FFA4)^10–12^, or a pair, (free fatty acid receptors 1-3, FFA1-3)^13,14^, of arginine residues that act to co-ordinate the carboxylate function of the fatty acid. It is well appreciated that in each of these cases the carboxylate is key to ligand function because equivalent fatty acid esters and amides are not activators of the corresponding receptor^8^. To date only a single study has reported on significant efforts to define the mode of binding of MCFAs to GPR84^3^. Surprisingly, given that all currently recognised free fatty acid receptors contain a suitable charge partner for the carboxylate of the fatty acid, this study^3^ failed to identify an equivalent arginine or other positively charged residue that might play a similar role in GPR84, instead suggesting that the MCFA carboxylate may reach deep within the core of the receptor to interact with asparagine 104 (residue position 3.36)^3^. For orphan receptors to be formally paired with their endogenous ligand(s) requires not only that the suggested ligand(s) can activate the receptor but also that the ligands are able to do so at concentrations commensurate with their circulating or local tissue concentrations^15^. For example, it has been suggested that the orphan receptor GPR35 is a functional receptor for kynurenic acid^16^. However, the very low potency of this ligand at human GPR35 has led commentators to question the physiological relevance of this proposed pairing^17,18^. Although MCFAs are reported to activate GPR84 with micromolar potency^2^ there remain questions about whether such levels of MCFAs are generally present within the body. Moreover, measures of the potency of MCFAs derive generally from experiments performed following heterologous expression of GPR84^2,4^. Such experiments often overestimate true ligand affinity at a receptor due to features of receptor reserve and direct measures of affinity of the MCFAs for GPR84 are lacking.

Across the group of GPCRs activated by free fatty acids there is substantial evidence for multiple binding sites for both endogenously produced and synthetic ligands. Recent studies on FFA1 showed co-operative effects between a fatty acid and the synthetic, clinically-trialled partial agonist TAK-875^19^ and multiple binding pockets on this receptor have been confirmed in atomic level X-ray structures of the receptor^20^. Furthermore, although no equivalent crystal structures are yet available, functional studies have identified synthetic small molecule ligands that appear to bind to locations distinct from the short chain fatty acid binding sites on both FFA2^21,22^ and FFA3^23^, see^8^ for review. Finally, although limited in detailed characterisation, there are also studies consistent with the presence of multiple binding pockets in GPR84^3,24^.

Herein we address these issues for the purported MCFA-GPR84 pairing and in so doing demonstrate that, as expected for a free fatty acid receptor, an arginine residue, here located within the 2^nd^ extracellular loop (ECL2) of GPR84, acts as the putative charge partner for MCFAs to allow their recognition by the receptor. We also show that a series of other GPR84 agonist ligands with long hydrophobic tails and carboxylate bioisostere head groups^4,25,26^ bind the receptor in a manner overlapping with the MCFAs and, as such, these each act as ‘orthosteric’ agonists. By contrast, 3,-3′-diindolylmethane (DIM)^3,24,27^ (Fig. 1), previously shown to be an ‘allosteric’ activator of GPR84^2,3,24^, does not appear to bind to the GPR84 orthosteric site. DIM, and certain related molecules produce extensive increases in the measured potency of both MCFAs and other orthosteric agonists and this suggests that endogenously produced molecules acting in a similar way may result in significantly lower concentrations of MCFA being required to activate GPR84 than would have previously been predicted. Finally, we demonstrate that a group of GPR84 antagonist ligands^28^, although able to block signalling by both orthosteric and allosteric agonist ligands, do so non-competitively by binding at a location that is distinct from either of these agonist sites.

## Results

### Decanoic acid is an endogenous ligand that can activate GPR84

**Figure 2:**
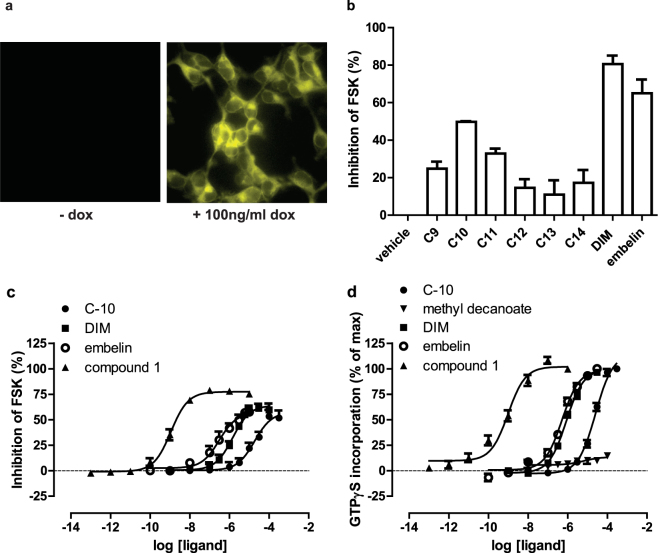
C10 and other ligands can activate GPR84. Flp-In T-REx 293 cells harboring FLAG-hGPR84-eYFP at the Flp-In T-REx locus were untreated (− dox) or maintained in the presence of doxycycline (100 ng/ml) for 24 hours. Such cells were imaged to detect the eYFP tag linked to the receptor (**a**). The capacity of fatty acids of chain length 9–14 (100 μM) to inhibit forskolin-stimulated cAMP levels in doxycycline-induced cells was assessed and compared to effects of embelin and DIM (**b**). C10, embelin, DIM and compound 1 each regulate cAMP levels in a concentration-dependent manner (**c**). Membrane preparations generated from doxycycline-induced cells were also used in [^35^S]GTPγS binding assays and each of C10, embelin, DIM and compound-1 promoted binding of [^35^S]GTPγS in a concentration-dependent manner (**d**). By contrast methyl decanoate was without effect (**d**).

We expressed a form of human (h)GPR84 (FLAG-hGPR84-eYFP) that incorporated both an N-terminal FLAG epitope-tag sequence and C-terminal enhanced Yellow Fluorescent Protein (eYFP) in Flp-In T-REx 293 cells. In such cells addition of the antibiotic doxycycline allows induced expression of DNA constructs in the Flp-In T-REx locus^29^. Imaging of such cells showed that the FLAG-hGPR84-eYFP construct was expressed effectively (Fig. 2a). GPR84 is recognized to interact selectively with pertussis toxin-sensitive, G_i_-family G proteins^4,8^. In the initial de-orphanization study on GPR84 Wang and colleagues^2^ showed MCFAs with chain length C9-C14 were able to activate GPR84. Initially, we used this same group of fatty acids and demonstrated that when using 100 μM of each MCFA, decanoic acid (C10) produced the largest effect as assessed by measuring the capacity of the ligands to inhibit forskolin-amplified levels of cAMP in cells expressing FLAG-hGPR84-eYFP (Fig. 2b). As positive controls in these studies we used two further ligands recognized to be agonists of GPR84; 2,5-dihydroxy-3-undecyl-2,5-cyclohexadiene-1,4-dione (embelin)^5^ (Fig. 1) and DIM (Fig. 1). Concentration-response studies showed both embelin (pEC_50_ = 6.7 +/− 0.3) and DIM (pEC_50_ = 5.9 +/− 0.1) to be substantially more potent than C10 (pEC_50_ = 4.7 +/− 0.2) (Fig. 2c). G_i_-coupled receptors are frequently highly effective in promoting binding of the guanine nucleotide analog [^35^S]GTPγS to receptor-associated G proteins^30^. Using this as an alternate end-point both embelin (pEC_50_ = 6.2 +/− 0.1) and DIM (pEC_50_ = 6.0 +/− 0.1) also promoted enhanced binding of [^35^S]GTPγS to membrane preparations of cells induced to express FLAG-hGPR84-eYFP (Fig. 2d). Once more these were both considerably more potent than C10 (pEC_50_ = 4.6 +/− 0.1). We also assessed a more recently reported GPR84 agonist, 2-(hexylthiol)pyridimine-4,6 diol (designated compound-1^25^, or ZQ16^26^, in previous reports) (Fig. 1). This was substantially more potent as an agonist in both the inhibition of cAMP (pEC_50_ = 9.0 +/− 0.1) (Fig. 2c) and [^35^S]GTPγS binding (pEC_50_ = 9.1 +/− 0.2) (Fig. 2d) assays. Importantly, methyl decanoate (Fig. 1), lacking the carboxylate function of C10, was completely inactive at GPR84 (Fig. 2d).

### DIM is a positive allosteric modulator of the potency of decanoic acid

**Figure 3:**
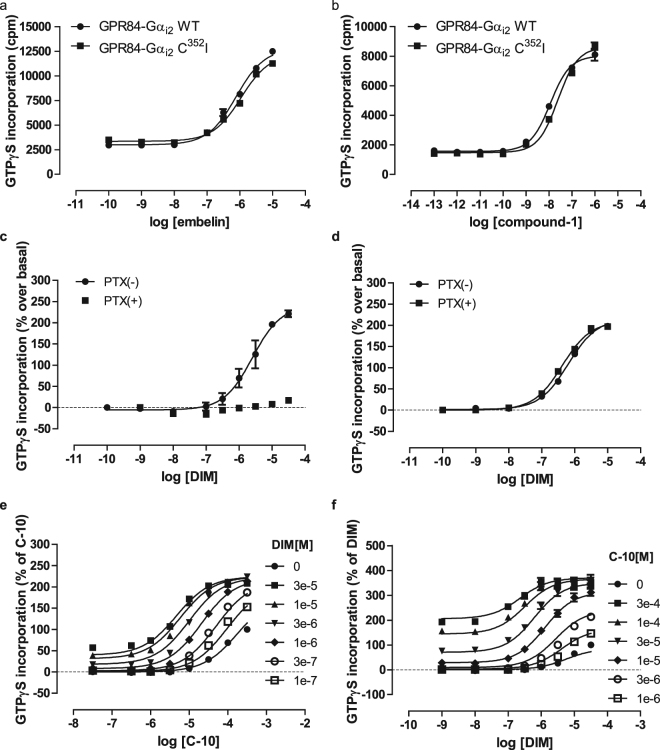
C10 and DIM display allosteric interactions in activating GPR84. Either a GPR84-Gα_i2_ fusion protein, or a pertussis toxin-resistant variant, GPR84-Cys^352^Ile Gα_i2_, was expressed in Flp-In T-REx 293 cells. Membrane preparations were then generated and used to assess the effect of varying concentrations of embelin (**a**) or compound-1 (**b**) on binding of [^35^S]GTPγS. Cells as above expressing (**c**), GPR84-Gα_i2_ or (**d**), GPR84-Cys^352^Ile Gα_i2_ were treated with or without pertussis toxin (PTX) for 24 h prior to membrane production. The effects of varying concentrations of DIM were then measured. How fixed concentrations of DIM affected the concentration-dependence of C10-induced stimulation of binding of [^35^S]GTPγS (**e**) and *vice versa* (**f**) was assessed. Details of fitting datasets akin to the examples shown in (**e**) and (**f**) to an operational model of allosteric modulation^46^ is provided in Table 1.

The only previous study^3^ designed to explore the mode of binding of ligands to GPR84 employed a fusion protein^31,32^ in which the G protein Gα_i1_ was linked in-frame to the C-terminal tail of GPR84. Therefore, we also generated a pair of related GPCR-G protein fusion constructs in which, because Gα_i1_ displays a restricted distribution pattern whilst Gα_i2_ is expressed ubiquitously, either wild type Gα_i2_ or a pertussis toxin-resistant variant of this, Cys^352^Ile Gα_i2_^33^, was linked in-frame to the C-terminal tail of GPR84. Following expression of either of the GPR84-Gα_i2_ fusion proteins in Flp-In T-REx 293 cells and membrane preparation the measured potency of each of embelin and compound-1 was indistinguishable between the two GPR84-G protein fusion constructs (Fig. 3a,b). Response to DIM was all but eliminated when using membranes generated from cells that had been induced to express the wild type GPR84-Gα_i2_ fusion protein and then pre-treated with pertussis toxin (Fig. 3c). In contrast, response to DIM was unaffected following pertussis toxin treatment of cells induced to express the pertussis toxin-resistant GPR84-Cys^352^Ile Gα_i2_ fusion protein (Fig. 3d). These studies indicate that following pertussis toxin treatment of cells expressing the GPR84-Cys^352^Ile Gα_i2_ fusion protein all agonist-induced binding of [^35^S]GTPγS reflects incorporation of nucleotide into the receptor-linked G protein α subunit. Using this system the ability of various concentrations of C10 to stimulate binding of [^35^S]GTPγS was enhanced by co-addition of increasing concentrations of DIM (Fig. 3e). As such, as well as a direct agonist, DIM acted as a positive allosteric modulator (PAM) of the potency of C10 (Fig. 3e, Table 1). As anticipated for such an allosteric effect, equivalent outcomes were also observed when the experimental protocol was reversed and effects of increasing concentrations of C10 on the potency of DIM were assessed (Fig. 3f, Table 1). Importantly, as well as defining that C10 and DIM likely bind to topographically distinct sites on GPR84, mathematical analysis of these datasets provided estimates of the binding affinity of the ligands to the GPR84-Gα_i2_ fusion protein. These predicted C10 to bind with very modest affinity (K_A_ range 0.17–0.53 mM), whilst DIM (K_A_ range 5.6–6.9 μM) was predicted to bind with more than 200 times greater affinity (Table 1).

**Table 1:** Estimation of binding affinities and the allosteric characteristics of pairs of GPR84 agonists.

Agonist^a^	Modulator^b^	logα	logβ	pK_A_^c^	pK_B_^d^
compound-1	DIM	2.1 ± 0.07	-0.2 ± 0.05	6.7 ± 0.06	5.2 ± 0.04
DIM	compound-1	1.8 ± 0.08	1.14 ± 0.03	5.3 ± 0.04	6.9 ± 0.07
compound-1	3a	1.75 ± 0.05	-0.1 ± 0.02	6.8 ± 0.03	5 ± 0.04
3a	compound-1	1.43 ± 0.08	1.2 ± 0.06	4.83 ± 0.06	7.2 ± 0.05
C-10	DIM	1.4 ± 0.1	0.4 ± 0.07	3.3 ± 0.08	5.2 ± 0.03
DIM	C-10	1.3 ± 0.1	1.2 ± 0.05	5.2 ± 0.06	3.8 ± 0.06
embelin	DIM	1.22 ± 0.05	0.8 ± 0.02	5.5 ± 0.03	5.15 ± 0.03
DIM	embelin	1.12 ± 0.04	1.0 ± 0.03	5.1 ± 0.03	5.7 ± 0.02

^a^Agonist is the compound used to generate concentration-response curve.

^b^Modulator is the compound used in defined concentrations.

^c^pK_A_ are values estimated for the agonist.

^d^pK_B_ are values estimated for the modulator.

Data are means ± SEM.

**Figure 4:**
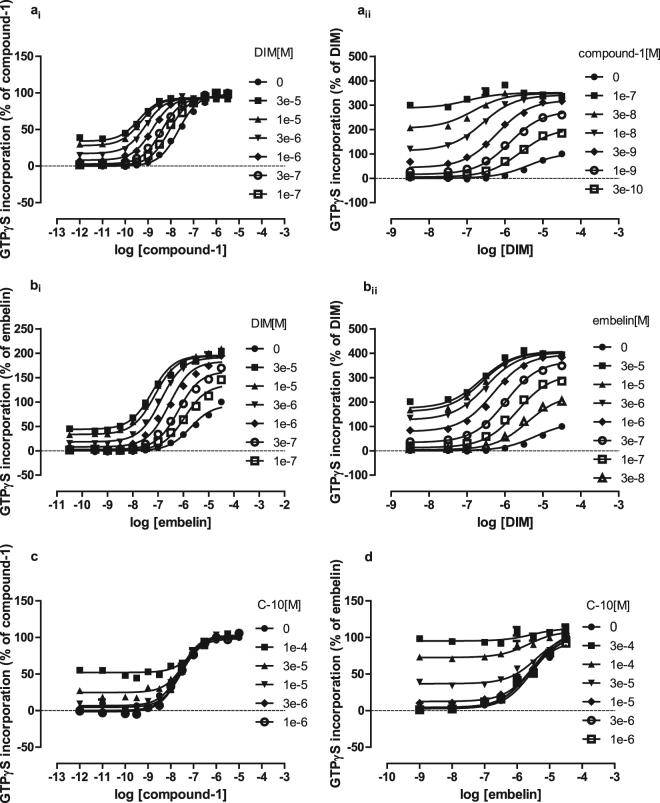
DIM also displays allosteric interactions with other orthosteric GPR84 agonists. Experiments were performed as in Fig. 3 to assess potential positive allosteric effects between DIM and compound-1 (**ai,ii**) and embelin (**bi,ii**). Similar studies assessed potential allosteric effects between C10 and either compound-1 (**c**) or embelin (**d**). Table 1 provides quantitative outcomes of fitting datasets akin to those shown in (**a**) and (**b**) to an operational model of allosterism.

We next assessed whether DIM would also affect the measured functional potency of either embelin or compound-1. For both pairings this was the case, with increasing concentrations of DIM enhancing the measured potency of compound-1 (Fig. 4a) and embelin (Fig. 4b) and *vice versa* (Fig. 4a,b). This demonstrated that both compound-1 and embelin also most likely bind to GPR84 at a different site(s) than DIM. Moreover, the binding affinity of DIM calculated from the PAM effects of each of embelin and compound-1 on potency of DIM is anticipated to be independent of the identity of the second ligand used in such studies and, indeed, in both these cases mathematical analysis of the datasets once more allowed assessment of affinity of DIM for the GPR84-Gα_i2_ fusion protein as being between 5 and 10 μM (Table 1). By contrast, co-addition studies using C10 with either compound-1 (Fig. 4c) or embelin (Fig. 4d) did not result in modulation of the potency of the ligands, and these results are consistent with C10, compound-1 and embelin sharing a single, common binding site.

### Analogs of DIM lose functional activity

**Figure 5:**
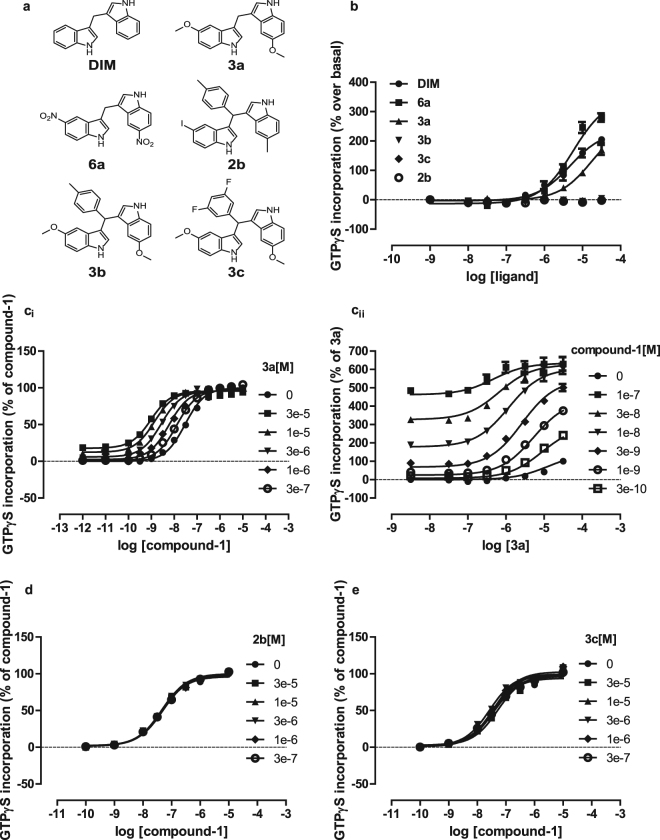
Certain DIM analogs lack affinity at GPR84. The ability of DIM and various analogs of DIM,(structures in **a**) to promote binding of [^35^S]GTPγS to membranes of Flp-In T-REx 293 cells expressing the GPR84-Gα_i2_ fusion protein was assessed (**b**). The effect of compound 3a on the concentration-response curve to compound-1 (**ci**) and *vice versa* (**cii**) and the lack of effect of compounds 2b (**d**) and 3c (**e**) on the concentration-response curve to compound-1 are displayed.

We next studied a series of analogs of DIM^27^ (Fig. 5a). Although some of these, including compounds ‘3a’ and ‘6a’ also showed direct agonism of GPR84 (Fig. 5b) others, including ‘2b’, ‘3b’ and ‘3c’ (Fig. 5b) were inactive. Each of the inactive ligands tested contains a bulky substitution on the linker between the indole groups. Whilst compound ‘3a’ produced as large positive allosteric effects on the potency of the orthosteric agonist ligands as did DIM (Fig. 5c), increasing concentrations of either ‘2b’ (Fig. 5d) or ‘3c’ (Fig. 5e) were unable to effect the position of the concentration-response curves for the orthosteric agonists of GPR84, including compound-1. These data suggest that unlike compound ‘3a’, compounds ‘2b’ and ‘3c’ lack affinity for GPR84. Studies with a broader range of such analogs^24^ may help define the allosteric agonist binding pocket.

### Defining an orthosteric binding mode

**Figure 6:**
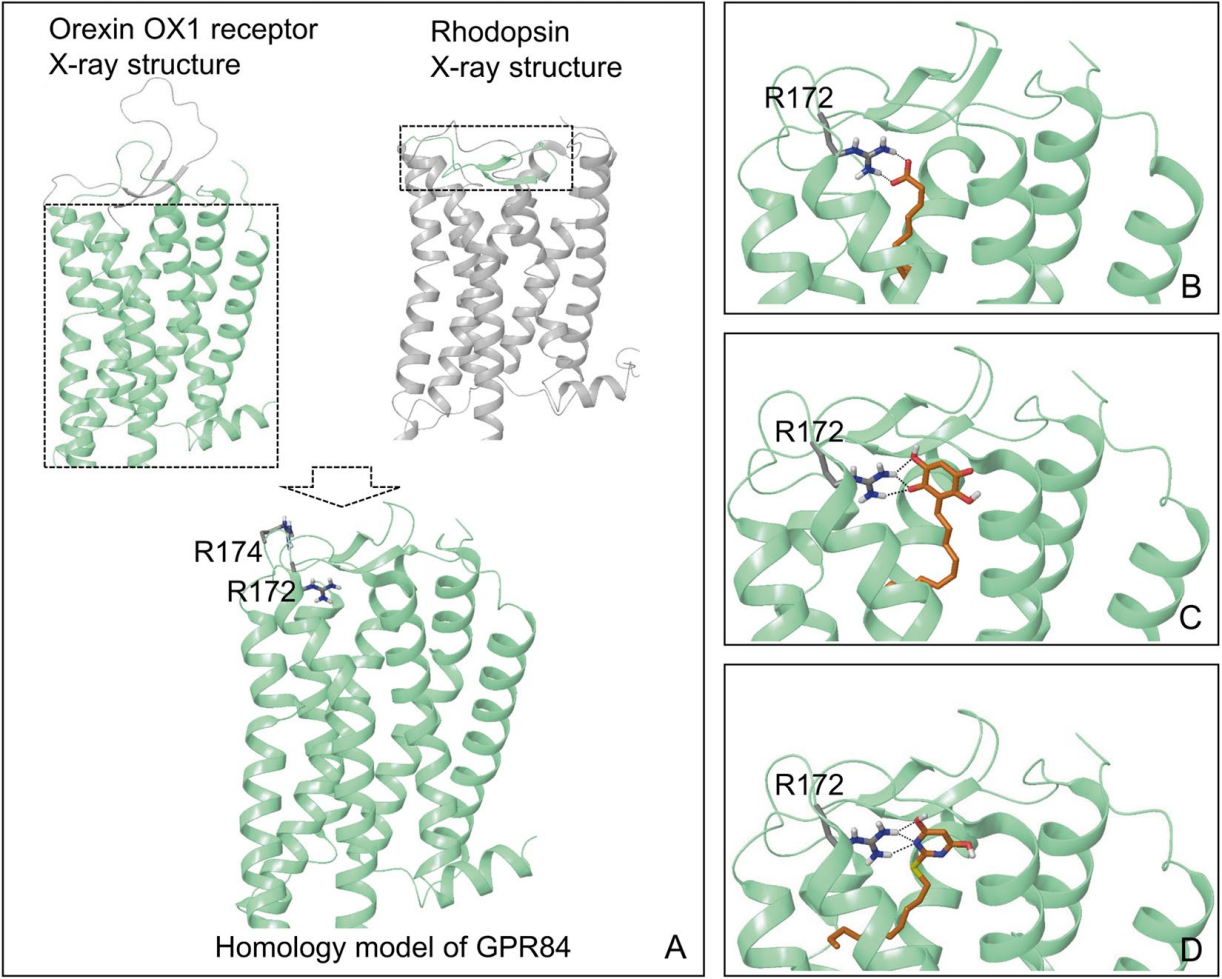
Homology model of GPR84 and the putative binding modes of orthosteric GPR84 agonists. (**A**) A GPR84 homology model was constructed based on a hybrid template involving the crystal structure of the helical bundle of the OX_1_ orexin receptor and the crystal structure of ECL2 of rhodopsin. Two arginines, Arg^172^ and Arg^174^ located within ECL2 are visualized. (**B**–**D**) The putative docking pose of C10 (**B**), embelin (**C**) and compound-1 (**D**), respectively is shown. The anchoring R^172^ within ECL2 is visualized. Hydrogen bonds or salt bridges are in dotted black lines.

**Figure 7:**
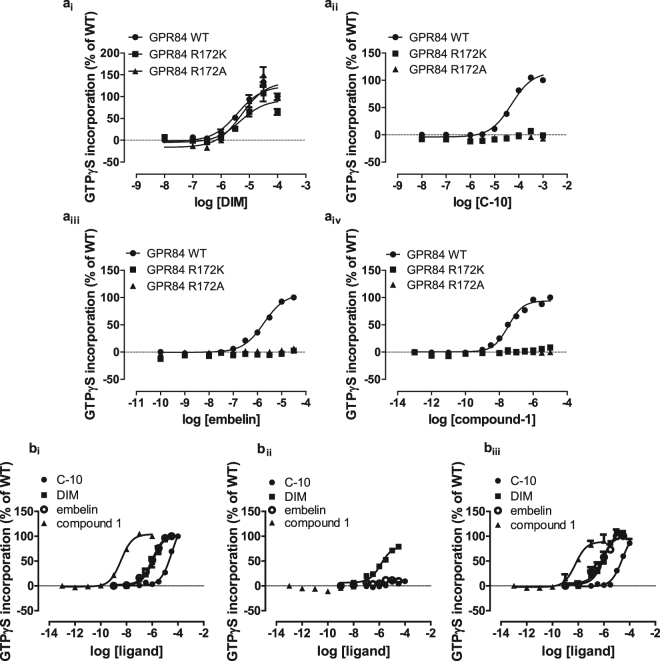
Conversion of arginine^172^ to alanine or lysine eliminates agonism of orthosteric agonists but not of DIM. (**a**) Membranes expressing either GPR84-Cys^352^IleGα_i2_, Arg^172^Ala GPR84-Cys^352^IleGα_i2_ or Arg^172^Lys GPR84-Cys^352^IleGα_i2_ were used in [^35^S]GTPγS binding studies to assess function of (**ai**) DIM, (**aii**), C10, (**aiii**) embelin and (**aiv**) compound-1. (**b**) Similar studies were performed on membrane of cells induced to express FLAG-hGPR84-eYFP (**bi**), FLAG-Arg^172^Ala hGPR84-eYFP (**bii**), FLAG-Arg^174^Ala hGPR84-eYFP (**biii**) to assess activity of C10, DIM, embelin and compound-1.

We selected, *a priori*, to designate the C10 binding site as the ‘orthosteric’ site because C10 is an endogenously produced ligand. To try to identify how a MCFA might interact with GPR84 we turned to homology modelling studies. Although GPR84 is not closely related to the other GPCRs that respond to either short chain (FFA2, FFA3) or long chain (FFA1, FFA4) fatty acids^8,9^, in each of the other cases the carboxylate of the fatty acid is co-ordinated by one or more arginine residues located at or near the extracellular surface^12–14^. A previous effort to model the structure of GPR84 and to predict the mode and orientation of binding of C10 developed a receptor homology model based on the atomic level, active-state structure of the β_2_-adrenoceptor^3^. These studies, however, did not identify a potential charge partner for the carboxylate of the fatty acid near the extracellular surface and, indeed, concluded that the carboxylate of C10 was pointing downwards, deep into the cleft of the seven transmembrane domain core of the receptor and interacting with an asparagine located at position 104 in the primary amino acid sequence^3^. As we have highlighted recently^9^ GPR84 and the β_2_-adrenoceptor are only distantly related whilst, of the currently available atomic level GPCR structures, the orexin OX_1_ receptor^34^ has highest overall sequence identity (31%) with GPR84. We, therefore, generated and employed a new homology model of GPR84 based on the transmembrane domain architecture of the OX_1_ receptor (Fig. 6). However, because ECL2 of GPR84 is very similar in sequence to that of rhodopsin (Fig. 6a), with 43% residue identity over this region, we incorporated this knowledge into the modelling process. In the resulting hybrid model (Fig. 6a) Arg^172^ within ECL2 is pointing into the potential binding cavity within the helical bundle. This was not the case when Nikaido *et al*.,^3^ employed the β_2_-adrenoceptor-based model, probably because the ECL2 sequences of the β_2_-adrenoceptor and GPR84 have no relatedness. This new model, therefore, provided the hypothesis that Arg^172^ might co-ordinate the carboxylate moiety of MCFAs, as well as the bioisosteric headgroups of embelin and compound-1. To assess this we mutated Arg^172^ to Ala within the setting of the GPR84-Cys^352^Ile Gα_i2_ fusion protein. Now, although the allosteric agonist DIM retained full activity at this mutant (Fig. 7ai), no response was produced by C10, embelin or compound-1 (Fig. 7aii–iv). Retention of positive charge at this position by generating an Arg^172^Lys mutation within the GPR84-Cys^352^Ile Gα_i2_ fusion protein also eliminated response to each of C10, embelin and compound-1 without affecting response to DIM (Fig. 7a). Equivalent results were obtained in studies in which the Arg^172^Ala mutation was introduced into the FLAG-hGPR84-eYFP construct (Fig. 7b). By contrast, mutation to Ala of Arg^174^, located only 2 amino acids away from Arg^172^, but instead predicted to point away from the binding cavity (Fig. 6a), did not affect function or potency of any of DIM, C10, embelin or compound-1 (Fig. 7b). Based on these studies, we explored potential docking poses of each of C10, embelin and compound-1. These studies suggest that the hydrophobic tail of each of these ligands projects downwards towards and into the core of the receptor (Fig. 6B–D).

### Studies with GPR84 antagonists

**Figure 8:**
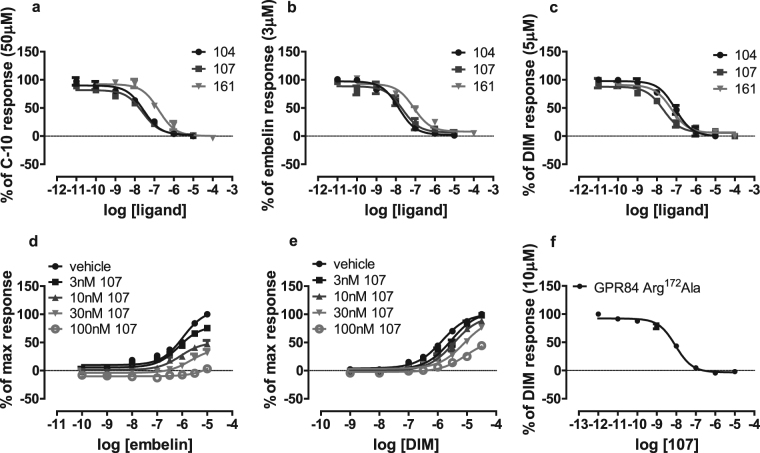
A number of GPR84 antagonists block agonist effects of both orthosteric and allosteric agonists. The ability of increasing concentrations of compounds 104, 107 and 161 to block agonism induced by EC_80_ concentrations of C10 (50 μM) (**a**), embelin (3 μM) (**b**) and DIM (5 μM) (**c**) were assessed. The ability of compound 107 to block activation of GPR84 by varying concentrations of embelin (**d**) or DIM (**e**) was also assessed. (**f**) Compound 107 inhibited DIM-induced binding of [^35^S]GTPγS in membranes of cells induced to Arg^172^Ala GPR84-Cys^352^IleGα_i2_.

Although the maintenance of agonist activity of DIM at Arg^172^Ala and Arg^172^Lys GPR84 provided confidence that these alterations did not simply generate a poorly organized and folded form of this receptor, we wished to explore this more fully. Labeguere *et al*.,^35^ have reported a series of compounds able to functionally antagonize GPR84. We explored the ability of three closely related exemplars from this series to limit [^35^S]GTPγS binding induced by EC_80_ concentrations of each of C10, embelin and DIM in membranes of cells expressing FLAG-hGPR84-eYFP. Compounds 104, 107 and 161 (9-(5-cyclopropyl-[1,2,4]oxadiazol-3-ylmethoxy)-2-((R)-1-[1,4]dioxan-2-ylmethoxy)-6,7-dihydro-pyrimido[6,1-a]isoquinolin-4-one; 2-([1,4]dioxan-2-ylmethoxy)-9-(3-phenylamino-prop-1-ynyl)-6,7-dihydro-pyrimido[6,1-a]isoquinolin-4-one; 2-((S)-1-[1,4]dioxan-2-ylmethoxy)-9-(tetrahydro-pyran-4-yl-methoxymethyl)-6,7-dihydro-pyrimido[6,1-a]isoquinolin-4-one, respectively) (Fig. 1) each did so, and in a concentration-dependent fashion, with 104 and 107 being more potent than 161 (Fig. 8a–c). Although able to block function of embelin, analysis of the effects of increasing concentrations of compound 107 on the position of the concentration-response curve for embelin showed that the antagonist decreased measured maximal effects of this agonist (Fig. 8d) rather than moving the measured EC_50_ of the agonist to higher concentrations as would be anticipated for a competitive and reversible receptor blocker. These features indicate that compound 107 blocks the effects of embelin in a non-competitive manner, likely by binding to a distinct site on GPR84. Moreover, at higher concentrations of 107 the maximal effect of DIM at GPR84 was also reduced (Fig. 8e), once more indicating a non-competitive mode of blockade of the agonism produced by DIM. Further support to define that compound 107 does not bind to the orthosteric agonist binding site was that compound 107 had equal potency to block DIM-stimulated binding of [^35^S]GTPγS to GPR84-Cys^352^Ile Gα_i2_ and to the Arg^172^Ala GPR84-Cys^352^Ile Gα_i2_ fusion proteins (Fig. 8f).

**Figure 9:**
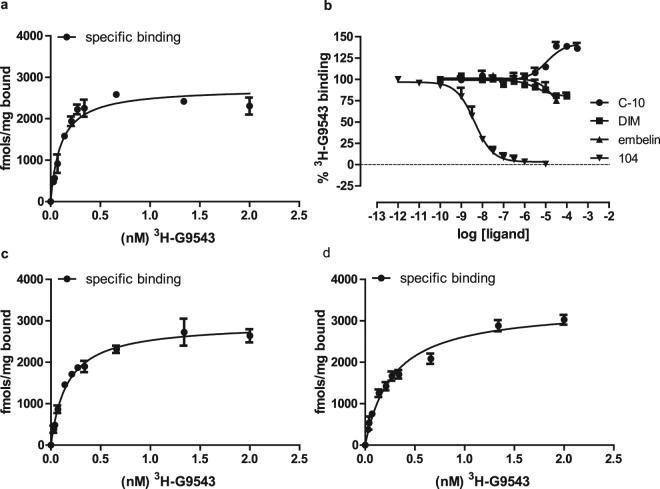
The antagonist [^3^H]G9543 binds GPR84 with high affinity but not at the orthosteric or allosteric agonist binding sites. Increasing concentrations of [^3^H]G9543 in the absence or presence of 1 µM compound 104 were assessed for their ability to bind to membrane preparations expressing wild type GPR84 (**a**). Specific binding of [^3^H]G9543, i.e. total binding minus that detected in the presence of 1 µM compound 104 is shown. Similar experiments were performed on membranes expressing Arg^172^Ala GPR84 (**c**) or Arg^174^Ala GPR84 (**d**). The ability of varying concentrations of compound 104, C10, embelin, and DIM to compete for binding of [^3^H]G9543 to GPR84 was also assessed (**b**).

We next studied the binding characteristics of a radiolabelled form of a GPR84 antagonist [^3^H]G9543 that is chemically related to compounds 104, 107 and 161 (Fig. 9). [^3^H]G9543 bound to membranes expressing FLAG-hGPR84-eYFP with high affinity (K_d_ = 0.24 +/− 0.07 nM) and in a manner consistent with a single binding site (Fig. 9a). This was also the case for the GPR84-Cys^352^Ile Gα_i2_ fusion protein (K_d_ = 0.26 +/− 0.02 nM). Binding of [^3^H]G9543 to the GPR84-Cys^352^Ile Gα_i2_ fusion protein was competed fully and effectively by the antagonist compound 104 (Fig. 9b) with pK_i_ of compound 104 assessed as 8.73 +/− 0.1. By contrast, neither C10 nor DIM was able to compete effectively with [^3^H]G9543 to bind GPR84 (Fig. 9b), and this was also the case for embelin (Fig. 9b). This suggests that [^3^H]G9543 and, by extension, compound 104 binds to a further site that is topographically distinct from that occupied by C10 and embelin and also from that occupied by DIM. This further indicates separation of the agonist and antagonist binding sites and that these GPR84 antagonists block agonist function at GPR84 in a non-competitive fashion. In further support for this model, binding affinity of [^3^H]G9543 was not altered substantially at the Arg^172^Ala GPR84-eYFP (K_d_ = 0.15 +/− 0.01 nM) (Fig. 9c) or, indeed, the Arg^174^Ala GPR84-eYFP (K_d_ = 0.25 +/− 0.06 nM) (Fig. 9d) variants.

## Discussion

Potential endogenous activating ligands have been suggested for a number of GPCRs that officially remain designated as ‘orphans’^1,5,7^. However, in many of these cases the suggested ligands have previously been paired with more certainty with other members of the GPCR superfamily, and/or the reported potency of the suggested ligand is modest at the receptor in question^1^. Moreover, in such cases there is generally also little known about the predicted mode of binding of the ligand to the receptor. Clearly such issues do not exclude the possibility that a physiologically relevant pairing has been uncovered, but does highlight that further and more detailed studies are generally required to provide further support or confirmation.

GPR84 is not closely related to either of the groups of previously defined free fatty acid-responsive GPCRs, FFA1-3 and FFA4. However, a feature shared by FFA1-4 is that each possesses at least one arginine residue located close to the extracellular face of the receptor that acts as the charge partner for the carboxylate moiety of fatty acid ligands^8,9^. Earlier studies showed that wild type GPR84 failed to respond to decylamine^3^ and herein we confirm the importance of the carboxylate of C10 to activation of the receptor because methyl decanoate was also unable to act as an agonist at GPR84. Previous mutational studies^3^ did not identify a positively charged amino acid that could act to co-ordinate the carboxylate of MCFAs, but instead suggested that the carboxylate might interact with an asparagine residue which modelling studies indicated to be deep within the central pocket created by the architecture of the seven transmembrane helices^3^. The homology model developed in these studies^3^ was based on the atomic level structure of a G protein-bound, active state of the β_2_-adrenoceptor. However, the β_2_-adrenoceptor has very limited sequence similarity to GPR84. In recent times the expansion of available atomic level structures of different GPCRs means that potentially better starting points for homology modelling can be selected from receptors that have higher levels of sequence similarity. Of the currently available structures, the orexin OX_1_ receptor^34^ is most closely related to GPR84, and using this as a template to model the transmembrane domain architecture, in concert with recognition that the sequence of ECL2 of GPR84 is highly homologous to that of rhodopsin, for which atomic level structures are known, allowed us to build a novel homology model^9^ that predicted that Arg^172^, within ECL2, might provide a charge partner for the fatty acid carboxylate. Indeed, this prediction was supported. Alteration of this residue to alanine completely eliminated receptor activation by C10. Moreover, a number of other GPR84 agonists in which an extended alkyl chain is linked to a head group that can be considered as a bioisostere of the fatty acid carboxylate all also lacked ability to activate Arg^172^Ala GPR84. Interestingly, all of these agonists also lost detectable activity when Arg^172^ was converted to Lys, indicating that the position of the positive charge is just as vital as its preservation. The size and shape of the positive charge of Lys is quite different to Arg and the modelling studies also suggested the Arg to be engaged in a network of interactions with close-by aromatic residues that help define the conformation and location of the amino acid side chain. Hence, as Lys would be unlikely to form the same contacts and therefore organize the binding pocket in an equivalent manner, it is not surprising that it is unable to substitute in this regard.

GPR84 antagonist ligands have, to date, been described in detail only in the patent literature^35^, but the antagonist GLPG1205 did enter a clinical trial for the treatment of ulcerative colitis^28^. Despite being safe and tolerable GLPG1205 did not show efficacy in patients with ulcerative colitis after 12 weeks of chronic dosing. Whilst antagonist compounds 104, 107 and 161, that derive from the same patented series as GLPG1205, were able to inhibit the function of both C10 and embelin in a concentration-dependent manner, binding studies performed using a further related radiolabelled antagonist [^3^H]G9543 indicated that affinity for this ligand was not lost by mutation of Arg^172^. Moreover, C10 was unable to effectively compete with [^3^H]G9543 for binding to GPR84. This suggests that rather than acting as orthosteric competitive antagonists these compounds block agonist function at GPR84 in a non-competitive fashion. Indeed, direct analysis indicated this to be the case in that whilst orthosteric agonist EC_50_ was little affected by increasing concentrations of compound 107, ongoing reduction in maximal agonist effects were observed with increasing concentrations of this antagonist.

As recently indicated by others^24^, DIM acted as an allosteric agonist at GPR84, and retained full function as an agonist at both Arg^172^Ala and Arg^172^Lys GPR84, where responses to C10 and other orthosteric agonists were lost. Moreover, as well as an allosteric agonist DIM acted as a highly effective PAM of the agonist actions of each of C10 and other orthosteric GPR84 agonists including embelin and compound-1. As anticipated from this, C10 and the other orthosteric agonists produced reciprocal effects on the potency of DIM. This may be important in relation to activation of GPR84 *in vivo*. Affinity estimates for DIM at GPR84, based on mathematical analysis of the positive allosteric effects of C10 and other orthosteric ligands on DIM, were in the region of 5–10 μM. This approach can provide estimates of ligand affinity at a GPCR whereas direct measures of agonist potency are often difficult to relate directly to ligand affinity if the target receptor is expressed heterologously in a cell line due to the potential for high level receptor expression resulting in a receptor reserve for function.

DIM can be produced *in vivo* by metabolism of the natural product indole-3-carbinol and following oral dosing to women of up to 1000 mg of indole-3-carbinol, plasma levels of DIM peaked within 2 hours at between 500–600 ng/ml (approximately 2 μM). However, this is rapidly cleared^36^. At peak levels, this is a sufficiently high level of DIM to partially activate GPR84 directly. Potentially of importance, however, the substantial positive allosteric effects of C10 on the observed potency of DIM indicated that at high levels of C10 or other MCFAs the potency of DIM to activate GPR84 would be some 20 fold higher than in the absence of C10. An effect of DIM could be manifest, therefore, at much lower concentrations, either arising from lower dosing with indole-3-carbinol or that the period of time before clearance of DIM to a level below that required for activation of GPR84 would be extended significantly. Perhaps more directly relevant to the *in vivo* situation, the detected low potency of MCFAs when studied in isolation would be increased into a range more akin to circulating concentrations of MCFAs. Although present in high levels in tropical oils such as coconut oil and palm kernel oil, levels of C10 in human plasma are generally low^37^, routinely below 0.5 μM. This is towards the limit of the range anticipated to be able to activate GPR84 when added in isolation. However, in the presence of 10 μM DIM this concentration of C10 would be anticipated to be sufficient to cause half-maximal activation of GPR84.

The concept that endogenously produced molecules may act as allosteric regulators of GPCRs, rather than uniquely acting as orthosteric ligands, is not entirely novel. Indeed, this concept has been reviewed recently^38^. Clearly many specific ions can act as allosteric regulators of agonist function at a broad range of GPCRs but, generally, concentrations of key ions are controlled within very strict limits. More interesting examples of endogenous allosteric regulators noted by^38^ include a variety of lipids and lipid-derived mediators, and thus it remains possible that MCFAs are acting at GPR84 as allosteric regulators rather than the true orthosteric activators of this receptor. While other means to generate the observed allosteric interactions between C10 and DIM can be envisaged, including the potential existence of dimeric forms of GPR84^39^, the most simple explanation for this and for maintenance of agonist function of DIM at Arg^172^Ala GPCR is that these two classes of agonists bind to distinct sites on a single receptor molecule. Similar reasoning supports that DIM must also bind at a site distinct from the GPR84 antagonists used herein. As for C10, agonist function of DIM was blocked by the GPR84 antagonists in a concentration-dependent manner and in a manner consistent with non-competitive interactions. Moreover, as for C10, DIM was unable to compete effectively with [^3^H]G9543 for binding to GPR84. These results are entirely consistent with the hypothesis that at least three spatiotemporally distinct ligand binding sites can be identified on GPR84. Recent structural studies suggest that this should not be entirely surprising. For example a recent atomic level structure of the protease activated receptor 2 (PAR-2) indicated ways in which multiple chemical series of blockers of a receptor may bind to non-overlapping sites^40^, whilst intracellular binding sites for antagonists of a number of chemokine receptor subtypes have recently been identified^41,42^. Clearly an atomic level structure of GPR84 is not available, but even without such information, mutagenesis studies should now start to help unravel the likely modes of binding of these distinct classes of pharmacological regulators of GPR84. Although the GPR84 antagonist GLPG1205 did not show efficacy in patients suffering from ulcerative colitis^28^ there remains considerable interest in targeting GPR84 in conditions that range from reflux esophagitis^43^, via cognitive decline^44^, to neuropathic pain^45^. Greater insights into the biology and pharmacological behaviour of ligands at GPR84 will assist in efforts to do so.

## Methods

### Materials

Medium chain fatty acids, methyl decanoate, embelin and DIM were from Sigma-Aldrich. Compound-1 and the GPR84 antagonist compounds 104 (9-(5-cyclopropyl-[1,2,4]oxadiazol-3-ylmethoxy)-2-((R)-1-[1,4]dioxan-2-ylmethoxy)-6,7-dihydro-pyrimido[6,1-a]isoquinolin-4-one), 107 (2-([1,4]dioxan-2-ylmethoxy)-9-(3-phenylamino-prop-1-ynyl)-6,7-dihydro-pyrimido[6,1-a]isoquinolin-4-one) and 161 (2-((S)-1-[1,4]dioxan-2-ylmethoxy)-9-(tetrahydro-pyran-4-yl-methoxymethyl)-6,7-dihydro-pyrimido[6,1-a]isoquinolin-4-one) (Fig. 1) were synthesized as in^25,35^. The analogs of DIM (Fig. 5) were a gift from Dorota Maciejewska, Department of Organic Chemistry, Medical University of Warsaw, Poland. [^35^S]GTPγS was from PerkinElmer Life Sciences. Tissue culture reagents were from Invitrogen and molecular biology enzymes and reagents from New England BioLabs. Polyethylenimine (PEI) [linear poly(vinyl alcohol) (MW-25000)] was from Polysciences (Warrington, PA). All other experimental reagents were from Sigma-Aldrich unless indicated otherwise.

### Plasmids and mutagenesis

A FLAG epitope (amino acid sequence DYKDDDDK) was introduced at the N-terminal end of human GPR84 cDNA by PCR using the following primers: sense, 5′ GCGGGATCCGCCACCATGGACTACAAGGACGACGATGATAAGTGTTGGAACAGCTCTGACGCC 3′, and antisense: 5′GTGGCGGCCGCGATGGAGCCTATGGAAACTCC 3′. The resulting cDNA was subsequently cloned in-frame into the BamHI and NotI sites of an eYFP-pcDNA5/FRT/TO plasmid yielding the final N-terminal epitope and C-terminal fluorescent protein-tagged construct FLAG-humanGPR84-eYFP-pcDNA5/FRT/TO.

FLAG-humanGPR84-Gα_i2_ fusion proteins were constructed by replacing eYFP within the FLAG-humanGPR84-eYFP-pcDNA5/FRT/TO construct with sequence corresponding to Gα_i2_ or Cys^352^Ile Gα_i2_ using NotI and XhoI restriction enzymes. Site-directed mutagenesis to generate point mutants of GPR84 was performed according to the QuikChange method (Stratagene, Cheshire, UK). The identity of all constructs was verified by nucleotide sequencing.

### Cell culture, transfection and generation of cell lines

Flp-In TREx 293 cells (Invitrogen) were maintained in Dulbecco’s modified Eagle’s medium without sodium pyruvate (Invitrogen), supplemented with 10% (v/v) fetal calf serum, 1% penicillin/streptomycin mixture, and 10 μg/ml blasticidin at 37 °C in a 5% CO_2_ humidified atmosphere. To generate Flp-In TREx 293 cells able to inducibly express the various GPR84 receptor constructs the cells were transfected with a mixture containing the desired cDNA in pcDNA5/FRT/TO vector and pOG44 vector (1:9) by using 1 mg/ml PEI (MW-25000). Cells were grown until 60 to 80% confluent then transfected with 8 μg of required plasmid DNA and PEI (ratio 1:6 DNA/PEI), diluted in 150 mM NaCl, pH 7.4. After incubation at room temperature for 10 min, the mixture was added to cells. After 48 h the medium was changed to medium supplemented with 200 μg/ml hygromycin B to initiate the selection of stably transfected cells. After isolation of resistant cells, expression of the appropriate construct from the Flp-In TREx locus was induced by treatment with up to 100 ng/ml doxycycline for 24 h.

### HTRF-based cAMP inhibition assays

All cAMP experiments were performed using Flp-In T-REx HEK293 cells induced to express the receptor of interest. Experiments were carried out using a homogenous time-resolved FRET-based detection kit (CisBio Bioassays; CisBio, Codolet, France) according to the manufacturer’s protocol. For the assay cells were plated at 5000 cells/well in low-volume 384-well plates. The ability of agonists to inhibit 1 μM forskolin-induced cAMP production was assessed following a co-incubation for 30 min with agonist compounds. Reactions were stopped according to the manufacturer’s instructions and the output was measured by with a PHERAstar FS plate reader (BMGLabtech, Aylesbury, UK).

### Membrane preparation

Membranes were generated from Flp-In T-REx HEK293 cells treated with 100 ng/mL doxycycline to induce expression of the receptor construct of interest. Cells were washed with ice-cold phosphate-buffered saline, removed from dishes by scraping and centrifuged at 3000 rpm for 5 min at 4 °C. Pellets were resuspended in TE buffer (10 mM Tris-HCl, 0.1 mM EDTA; pH 7.5) containing a protease inhibitor mixture (Roche Applied Science, West Sussex, UK) and homogenized with a 5 ml hand-held homogenizer. This material was centrifuged at 1500 rpm for 5 min at 4 °C and the supernatant was further centrifuged at 50000 rpm for 45 min at 4 °C. The resulting pellet was resuspended in TE buffer and protein content was assessed using a BCA protein assay kit (Pierce, Fisher Scientific, Loughborough, UK).

### [^35^S]GTPγS incorporation assay

Initially, 5 μg of generated membrane protein was pre-incubated for 15 min at 25 °C in assay buffer (20 mM HEPES, 5 mM MgCl_2_; 160 mM NaCl; 1 μM GDP; 0.05% fatty acid-free bovine serum albumin; pH 7.5) containing the indicated ligand concentrations. In experiments designed to assess potential allosteric interactions between DIM and related molecules and either C10 or the other ‘orthosteric’ agonist the compounds were added at the same time to the membrane preparation. The reaction was then initiated with addition of [^35^S]GTPγS (50 nCi per tube), and the reaction was terminated after 45 minutes incubation at 30 °C by rapid filtration through GF/C glass filters using a 24-well Brandel cell harvester (Alpha Biotech, Glasgow, UK). Unbound radioligand was removed from filters by three washes with ice-cold phosphate buffered saline (pH 7.4) and filters were dried for 2–3 h at room temperature. Dried filters were added to 3 mL of Ultima Gold^TM^ XR (PerkinElmer Life Sciences, Beaconsfield, UK) and [^35^S]GTPγS binding was determined by liquid scintillation spectrometry.

### Radioligand binding assay

[^3^H]G9543 is an analog of compounds 104, 107 and 161 with the same characteristic 2-(substituted-alkoxy)-9-substituted-6,7-dihydro-pyrimido[6,1-a]isoquinolin-4-one chemotype. Assays were carried out either with increasing concentrations (for saturation binding) or respective K_d_ concentrations (for displacement assays) of [^3^H]G9543, binding buffer (phosphate-buffered saline with 0.5% fatty acid free bovine serum albumin; pH 7.4), and the indicated concentrations of test compounds (for displacement assays) in a total assay volume of 500 µL in glass tubes. Binding was initiated by the addition of membranes (5 µg of protein per tube). All assays were performed at 25 °C for 1 h before termination by the addition of ice-cold phosphate-buffered saline and vacuum filtration through GF/C glass filters using a 24-well Brandel cell harvester (Alpha Biotech, Glasgow, UK). Each reaction tube was washed three times with ice-cold PBS. The filters were allowed to dry for 2–3 h and then placed in 3 ml of Ultima Gold^TM^ XR. Radioactivity was quantified by liquid scintillation spectrometry. Specific binding was defined as the difference between binding detected in the presence and absence of 1 µM compound 104.

### Data analysis

All data are presented as means ± SEM of at least three independent experiments. Data analysis and curve fitting was carried out using the GraphPad Prism software package version 5.0b (GraphPad, San Diego). For functional assays the concentration-response data were plotted on a log axis, with the untreated vehicle control plotted at 1 log unit lower than the lowest ligand concentration, and fitted to a three parameter sigmoidal curve with the Hill slope constrained to equal 1. In case of inhibition experiments with antagonists an equivalent analysis was followed to fit an inverse sigmoidal curve. To perform the statistical analysis of curve parameters, data from multiple experiments were fitted independently and resulting curve fit values were analysed with indicated tests. For radioligand binding data, saturation binding curves were generated by fitting the specific binding, which was obtained by subtracting non-specific from total binding, to a one site specific binding model that allows calculation of K_d_ values for the radioligand at wild type and mutant receptors. To determine affinity of unlabelled ligands, data obtained in displacement assays were fit to an inverse three parameter sigmoidal curve constrained by radioligand affinity and concentration to allow for K_i_ calculation.

To quantify the extent of allosteric interaction between pairs of ligands, data were analysed using an operational model of allosteric modulation described previously^46^. The general version of this model can be depicted by the following equation:

$$E=\frac{{E}_{m}{({\tau }_{A}[A]({K}_{B}+\alpha \beta [B])+{\tau }_{B}[B]{K}_{A})}^{n}}{{([A]{K}_{B}+{K}_{A}{K}_{B}+[B]{K}_{A}+\alpha [A][B])}^{n}+{({\tau }_{A}[A]({K}_{B}+\alpha \beta [B])+{\tau }_{B}[B]{K}_{A})}^{n}}$$

where, E is the pharmacological effect, [A] and [B] are the orthosteric and allosteric compound concentrations at equilibrium, respectively; K_A_ and K_B_ are the equilibrium dissociation constants of the orthosteric and allosteric ligands respectively, denoting the binding affinities of the two ligands to the receptor; α is the binding cooperativity factor denoting the magnitude and direction of the allosteric effect on binding affinity of the orthosteric agonist to the receptor, β is the activation cooperativity factor representing the measure of the allosteric effect on orthosteric efficacy. τ_A_ and τ_B_ represent the intrinsic activity of the orthosteric and allosteric ligand, respectively. E_m_ is the maximal possible system response and n denotes the slope factor of the transducer function. In case of global fitting of the allosterism data through this equation, E_m_ and n values were always constrained and other parameters (K_A_, K_B_,τ_A_, τ_B_, α, β) were estimated.

### Molecular modelling

The OX_1_ receptor and rhodopsin crystal structures with the PDB codes of 4ZJ8 and 2Z73, respectively, were used as templates to create a hybrid model of GPR84. In particular, the helical bundle from the OX_1_ receptor crystal structure was assembled with the second extracellular loop from the rhodopsin crystal structure and the combined template was used in modelling of GPR84. The homology model of GPR84 was generated with the Prime 3.8 module of the Schrodinger software^47^ with the default energy-based method^48^. The obtained homology model was refined in the MacroModel 10.6 module of the Schrodinger software package^47^ using short molecular mechanics and dynamics optimization. Molecular docking to the GPR84 model was conducted using the InducedFit module^49,50^ of the Schrodinger software^47^. The receptor grid was defined around Arg^172^. The side chain trimming was applied for Tyr^69^ and Phe^335^ that largely occluded the binding cavity. The best docking pose was chosen based on the docking energy and interactions with Arg^172^. Figures of the molecular models were generated with Maestro 9.9 of the Schrodinger software package^47^.

### Data availability

The datasets generated and analysed during the current study are available from the corresponding author on reasonable request.
